# “The credential did make a difference”: eating disorder treatment with ANZAED credentialed clinicians: carer perspectives

**DOI:** 10.1186/s40337-025-01383-y

**Published:** 2025-08-28

**Authors:** Honor Sinclair, Janet Conti, Madalyn McCormack, Gabriella Heruc, Katarina Prnjak, Rebecca Barns, Phillipa Hay

**Affiliations:** 1 School of Psychology, Western Sydney University, Penrith, Australia; 2 Translational Health Research Institute, School of Medicine, Western Sydney University, Locked Bag 1797, Penrith, 2751 Australia; 3 Eating Disorders and Nutrition Research Group, Translational Health Research Institute, School of Medicine, Western Sydney University, Penrith, Australia; 4 Graduate School of Health, University of Technology Sydney, Sydney, Australia; 5 Mental Health Services, Campbelltown Hospital SWSLHD, Campbelltown, Australia

**Keywords:** Eating disorders, Credential, Carers, Qualitative, Experience, Perception

## Abstract

**Background:**

The Australia & New Zealand Academy for Eating Disorders (ANZAED) has developed the Credential that recognises the qualifications, knowledge and professional development training, including continuing professional development (CPD), that comprise the minimum standard for the safe and effective delivery of treatment for an eating disorder. The current study sought to explore whether the anticipated benefits have translated into positive eating disorder treatment experiences with credentialed clinicians from carers’ perspectives.

**Methods:**

Thirteen carers of people living with an eating disorder (ED) were interviewed about their perceptions and experiences of ED treatment from clinicians awarded the ANZAED Eating Disorder Credential. Semi-structured interview transcripts were analysed with an inductive thematic analysis.

**Results:**

Three main themes with embedded subthemes were generated. Theme one explored treatment experiences prior to the launch of the Credential in November 2021. Theme two captured carers’ attitudes and perceptions of the Credential, including perceived benefits, other priorities, and suggestions for further improvement. The final theme explored carers’ treatment experiences with credentialed clinicians that were related to greater perceived knowledge and understanding and experiences of personalised therapeutic approaches to care.

**Conclusions:**

These findings hold important implications for future considerations of credentialing of ED clinicians and proposals for refinements of the ANZAED Eating Disorder Credential. Carers perceived the Credential to enhance quality of care and support, however, many noted that broader systemic issues were limiting its reach. Further efforts are needed to increase awareness and facilitate access to credentialed clinicians through key referral pathways.

**Supplementary Information:**

The online version contains supplementary material available at 10.1186/s40337-025-01383-y.

## Abstract (plain language summary)

Eating disorders (ED) are serious mental health conditions that require specialised care. The Australia & New Zealand Academy for Eating Disorders (ANZAED) developed a credential to ensure that clinicians possess the necessary training and expertise to provide effective ED treatment. This study aimed to understand whether carers (i.e., family members or those supporting individuals with EDs) perceived treatment from clinicians with the ANZAED Eating Disorder Credential as beneficial for their loved ones. Thirteen carers were interviewed about their perspectives. The findings revealed that many carers felt clinicians with the Credential demonstrated a better understanding of EDs and provided more supportive, individualised care. However, carers also highlighted ongoing challenges despite the Credential, such as difficulty accessing care and other broader systemic barriers. The study suggests that while the Credential may enhance the quality of care, additional efforts are needed to improve accessibility and awareness of credentialed clinicians in treatment settings.

**Supplementary Information:**

The online version contains supplementary material available at 10.1186/s40337-025-01383-y.

## Introduction

Eating disorders (EDs) have some of the most serious health consequences of any psychiatric disorder, with anorexia nervosa yielding the greatest mortality rate [1, 2]. However, it has been reported that between 30 and 73% of Australians never receive treatment for their ED symptoms [3]. Further, there remains considerable variability in the continuity, quality and accessibility of ED treatment [4] including in Australia [5].

There are also serious implications for carers, such as heightened distress and burden, disrupted family relationships, and increased suicide risk [6–9]. Carers and families play a vital role in ED treatment as both key supports [10] and drivers of change [11] regardless of the patient’s age. However, few researchers have explored the effectiveness of family interventions in adults or sought out carer perspectives directly; as such, carers are not yet regarded as true partners in care [12] and the nuances of their experience have been left largely unexplored or misunderstood [1, 13].

### Eating disorder treatment credentialling

Throughout the literature, training and continuous supervision have been identified as primary solutions to overcome systemic barriers preventing effective treatment in health contexts [14–17]. Accreditation is known to simultaneously preserve internal training quality while providing external quality assurance to service users and other stakeholders [18]. However, there is limited research exploring the benefits, costs and limitations of accreditation in general [19, 20]. However, there has been considerable discourse about possible benefits of credentialling across varying disciplines (e.g., psychology, dietetics & nursing). Credentialling can be beneficial through preserving the reputation of a profession by maintaining an adequate standard of knowledge, training, skills and experience [21] Credentialling systems have been said to instill greater consumer confidence [22, 23] this may relate to the notion that the public tend to perceive and identify credentialed clinicians as individuals who have at least met minimum standards of competence. However, there are some concerns about credentialling in the literature, these include a lack of financial benefits [24], financial costs to the professional [25], and the disenfranchisement of sufficiently qualified and proficient professionals who do not meet credentialing requirements [26].

In the context of eating disorders, there is an evident need for training to allow clinicians to treat clients and their carers competently and confidently. In addition, supervisors may play an important role in supporting clinicians to adhere to evidence-based treatment protocols, work within multidisciplinary teams, ensure family engagement and minimise clinician anxiety. An eating disorder credential could be a means to formally recognise a professional’s knowledge, training, qualifications, and development activities relevant to treating eating disorders [27].

Some global bodies, such as the Royal College of Psychiatrists [28] and the International Association of Eating Disorder Professionals Foundation [29] have developed a credential to standardize ED treatment. However, these initiatives are either limited to a single discipline (i.e., medicine), are limited in their capacity to coordinate multidisciplinary teams of clinicians, and/or depend on individual initiatives to complete professional development, without clear public recognition. There also exists a paucity of research that explores the outcomes of these credentials, leaving their implications for ED treatment unclear.

### The Australian ED credential initiative

In accordance with core treatment considerations, the National Eating Disorders Collaboration (NEDC) conducted a consultation process with key stakeholders (i.e., people with lived experience of EDs, families, carers, researchers and health professionals) to develop a standard of training and practise in ED treatment across disciplines [30]. This led to the identification of key principles for best practice and predictions that the perceived benefits of a credentialing system (e.g., enhanced treatment quality and consistency and reduced wait times for help seekers) would surpass any perceived costs (e.g., financial costs to professionals) [31]. The NEDC and ANZAED collaborated to create and launch the ANZAED Eating Disorder Credential (the Credential) recognising dietitians and mental health professionals who possess the knowledge, experience, and training for the provision of safe and effective eating disorder care. To obtain the Credential, clinicians need to have 2 years of clinical practice and have completed a NEDC approved course of introductory and treatment provision training. Under the “sunset clause”, some early applicants were permitted to provide written evidence of prior experience, knowledge and skill development in each of the required areas instead of completing the specified training [32].Credentialed clinicians are then required to complete a minimum of 15 h of ED specific continuing professional development (CPD) per year and receive a minimum of 6 h of ED specific supervision per year to maintain the credential [33].

With the Credential now in practice and service users beginning to receive treatment from credentialed clinicians, it is crucial to explore whether these predicted benefits are being realised.

Furthermore, despite consensus around the need to include carers in all levels of treatment, from planning to evaluation, there is a gap between research and practice. As such, further research is required to explicitly explore treatment experiences with ED credentialed clinicians from a carer’s perspective. Thus, the current study sought to explore carers’ perceptions and ED treatment experiences with Credentialed Eating Disorder Clinicians. The aims of this research are to contribute to a greater understanding of carer experiences of this recent reform and explore implications for further refinements.

## Methods

### Design

This cross-sectional qualitative study employed a six-step inductive thematic analysis framework [34]. Qualitative data collected via semi-structured interviews was supplemented by quantitative demographic data to explore carer perceptions and treatment experiences with credentialed clinicians.

### Participants

Participants included a carer (n = 1) and parents (n = 12) of individuals who experienced ED treatment with a credentialed clinician. Only individuals over the age of 18 who were currently residing in Australia were eligible for the study. Table 1 provides details on the characteristics of the carers who participated in this study. Statistics with small sample sizes were not reported for confidentiality reasons.

**Table 1:** Characteristics of Participants with Carer Lived Experience

Characteristics	Descriptive statistic	
	Median	IQR
Age (years)	52	47–53.5
Length of treatment (months)	24	11–25.5

	N	Percent (%)
Gender		
Female	12	92.3
Location of primary residence		
Metropolitan	10	76.9
Marital status		
Married/Living as married	10	76.9
Employment		
Employed part time, full time or casually	11	84.6
Education level		
Tertiary	12	92.3
Currently experiencing symptoms	10	76.9
ED diagnosis of loved one		
Anorexia Nervosa	11	84.6

Most participants identified as female (92.3%) and the median age of the sample was 52 years (IQR = 47–53.5). Participants were dispersed across Australia, with the most populated states being New South Wales (38.5%), Queensland (23.1%) and Victoria (23.1%). The majority of participants reported tertiary education or higher (92.3%), were married (76.9%), living in metropolitan areas (76.9%) and employed either full-time (30.8%) or part-time (53.8%).

Most participants were carers of someone who had experienced anorexia nervosa (84.6%), other less common diagnoses included Avoidant/Restrictive Food Intake Disorder (ARFID) and Other Specified Feeding and Eating Disorder (OSFED). In general (i.e., including both credentialed & non-credentialed clinicians), treatment most commonly involved general practitioners (also known as family physicians;100%), dietitians (92.3%), psychologists (92.3%) and psychiatrists (69.2%). The most common Credentialed Eating Disorder Clinicians seen were psychologists (55.6%), psychiatrists (44.4%), dietitians (44.4%), while others (88.8%) included social workers, occupational therapists, nurse practitioners, GPs, mental health nurses, and counsellors. All participants’ loved ones received in person treatment from a credentialed clinician with 60% also reporting telehealth sessions.

Commonly reported forms of intervention (from all professionals) included Family Based Therapy (FBT; 84.6%), medical or psychiatric Treatment by doctors who have prescribing medical training (69.2%), Cognitive Behavioural Therapy (CBT; 61.5%), nutritional counselling (61.5%) and inpatient medical or psychiatric therapy (53.8%). Due to missing data, cumulative percentages do not equal 100%, some exceed 100% due to categories not being mutually exclusive.

### Procedure and materials

This study was granted ethics approval by the Western Sydney University Human Research Ethics Committee (HREC Approval Number *H15252*). Participants were recruited via email, social media advertisements via Australian eating disorder services (SWAN centre, Eating Disorders Families Australia (EDFA), Butterfly Foundation, Eating Disorders Queensland (EDQ), Eating Disorders Victoria (EDV), Inside Out Institute) and through ANZAED and NEDC membership databases. Recruitment occurred in three waves from March 2023 to July 2023, with two follow ups occurring following an initial recruitment drive. Interested individuals were directed to an online link to the participant information statement, consent form and the survey (see Additional File 1) to complete. This survey collected data from 68 carers and comprised a range of closed and open-ended self-report questions first developed by the authors (PH, JC, KP) about participant demographics, clinical characteristics of their loved one’s ED, and their experiences and perceptions of treatment with a credentialed clinician.

After completing the survey, carers were asked whether their loved one had received treatment from a Credentialed Eating Disorder Clinician. They were then given the option to leave their email address if they wished to arrange a time to complete the interview. Carers whose loved one had not seen a credentialed clinician were not eligible to take part in the interviews. Interviews were conducted in a semi-structured online format via zoom. Interviews were then de-identified and transcribed via Zoom and reviewed (RB or NO) and emailed to participants to review for accuracy and remove any further potentially identifiable information that they did not wish to be included in findings from this study. Data collection continued until data sufficiency was reached; that is, no new information was elicited after three consecutive interviews [35]. There was no significant difference between the participants who took part in both the survey and interview and those who took part in the survey only (see Additional File 2 for a table comparing the two groups).

The interviews consisted of open-ended questions prompting participants to elaborate on their survey responses regarding their loved one’s experiences in accessing and receiving care for their ED. Questions also explored carer perspectives regarding resources from the connect·ed website and treatment from a credentialed clinician. The final interview schedule can be found in Additional File 3.

### Data analysis

A qualitative approach was selected to capture the complexity of caring for someone experiencing an ED and the nuances of the treatment experience. Given the relative paucity of research in the area and the novelty of the Credential in Australia, an inductive thematic analysis was employed [34]; allowing researchers to capture the complexities of carers’ experiences of treatment with a credentialed clinician. In addition, inductive analysis enabled themes and patterns to be drawn directly from the data without imposing an existing code frame to extrapolate and examine themes, patterns and meanings from the qualitative data (36).

Analysis followed the six steps of Braun and Clarke’s [34, 36] thematic analysis. This involved first, familiarisation with the data (i.e., immersion & documenting impressions (step 1; HS), followed by the formation of a coding system to generate initial themes (step 2). The data was then indexed numerically (step 3) and organised to generate the initial themes (HS). Themes were then reviewed (HS, JC, PH; step 4) and defined, and named (HS, JC, PH, MM; step 5). Finally, the themes were refined and interpreted, and comparisons were made between themes in the final write up of the findings (step 6; all authors). See Additional File 4 for the researchers’ positioning statements.

## Results

**Fig. 1:**
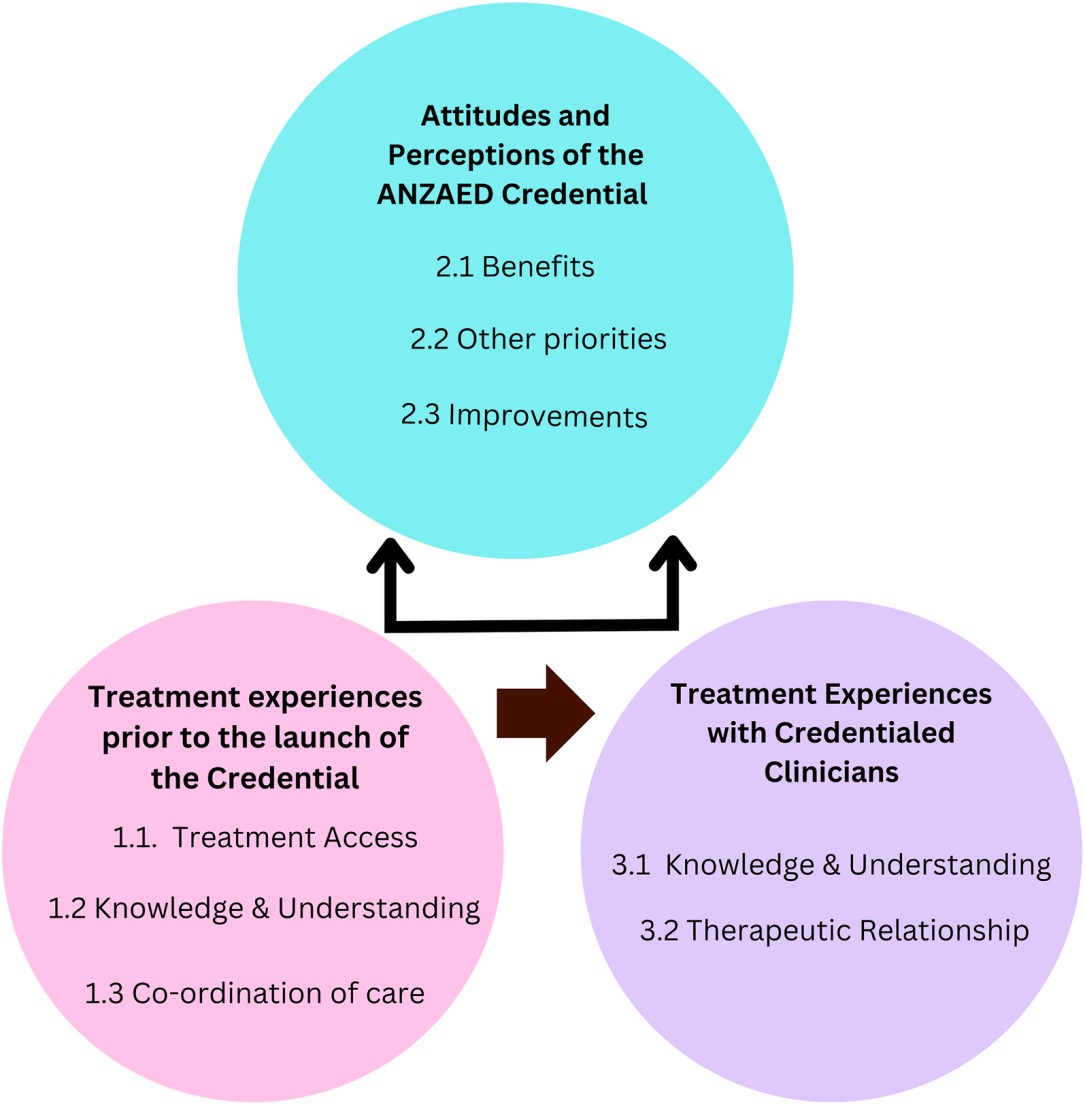
Thematic Map: inter-relationship of themes of carer perceptions of the credential and eating disorder treatment experiences

Analysis of carer’s experiences and perceptions of ED treatment from a credentialed clinician clustered around three main themes with embedded subthemes (Table 2). These themes and their inter-relationship are depicted in Fig. 1 and exemplar extracts for each of the subthemes are contained in Additional File 5.

**Table 2:** Carer experiences and perceptions of ED treatment from a credentialed clinician

*Theme 1: Treatment Experiences Prior to the Launch of the Credential*	
Subtheme 1.1: Treatment Access	
Subtheme 1.2: Knowledge and Understanding Prior to the Launch of the Credential	
Subtheme 1.3: Co-ordination of Care	
*Theme 2: Attitudes and Perceptions of the ANZAED Credential*	
Subtheme 2.1: Benefits	
Subtheme 2.2: Other Priorities	
Subtheme 2.3: Improvements	
*Theme 3: Treatment experiences with credentialed clinicians*	
Subtheme 3.1: Knowledge and Understanding	
Subtheme 3.2: Therapeutic Relationship

### Theme 1: Treatment experiences prior to the launch of the credential

Prior to seeing a credentialed clinician, most carers experienced significant difficulty accessing specialised ED treatment and lapses in support and prolonged periods of suboptimal therapeutic care were frequently reported.

#### Subtheme 1.1: Treatment access

Most carers reported unhelpful interactions and situations that they experienced throughout the treatment journey that they perceived to hinder effective treatment for their loved one. One of these included difficulties accessing early intervention, which carers related to clinicians’ reported failure to detect initial ED symptoms, insufficient resourcing, and limited availability of services. Despite most carers’ taking their loved one to a professional once they noticed early signs of an ED (mostly GPs, sometimes dietitians), many felt that their concerns were dismissed or unacknowledged. Often, it was not until symptoms became much more severe that clinicians began to recognise the ED symptoms, leading to diagnoses and appropriate referrals, which often included an inpatient admission. This demonstrated how for these participants, the ED had progressed to a critical state before specialist treatment was initiated. Indeed, one carer voiced, *“I’m angry that I was seeing something that nobody else could see, why didn’t we get on to it quicker?”* (P8). Implicit in this carer’s anger was their stance for their wisdom as a parent that had gone unacknowledged by likely inexperienced clinicians, requiring them to “*advocate”* (P8) for their child to receive the support they felt they needed from the outset of treatment.

Others noted lengthy wait times to see a psychologist as a major hinderance to recovery, inadvertently enabling time for the ED to worsen without necessary support: *“the waiting list is really, really, really long and the roots of the eating disorder grow deeper and deeper and deeper in the meantime” (P13).* Similarly, another carer reported contacting* “Thirteen referrals before we got on to a psychologist and dietitian who were able to take us”* (P7). Several carers also recounted feeling like they had *“fallen through the cracks”* (P5) when being passed from inpatient to outpatient, private or community services and hearing that their child’s presentation was too severe for one service or not severe enough for another, until they became unwell enough for another hospital admission, repeating the process. For example, one carer expressed that when they did find a psychologist with availability to see their child after several hospital admissions, *“they would say her BMI is too low there’s no point in engaging in conversation with her. So you really don’t know what to do. You can’t get into FBT (Family Based Therapy). You can’t get a psychologist” (P3).* Another carer noted that even when their child did meet hospital admission criteria (i.e., due to serious *“heart issues”),* the *“lack of resourcing”* prevented them from receiving a bed (P5). Indeed, this carer was told the hospital staff had checked “*five hospitals”* in the area and none of them had capacity, which they described as *“just gut wrenching”, “incredibly hopeless. And it was frightening. Because you know, what happened if he had a cardiac episode while I was at the shops or something”* (P5). According to this carer, *“that was the worst bit, just being told that the system couldn’t help us. That was just horrendous…And so, you end up in this holding pattern where life is just not progressing”* (P5).

These carer experiences highlight how barriers to care for an ED went beyond inexperienced clinicians to include systemic failures to adequately treat individuals with a stepped care approach to holistic ED care. A parallel process was then evident whereby this overwhelm of the system was overwhelming for carers who were left with a sense of hopelessness and reduced agency in their care of their loved one.

#### Subtheme 1.2: Knowledge and understanding prior to the launch of the credential

Once carers were able to access treatment, many questioned clinicians’ level of training or understanding of EDs, due to the time it took to make diagnoses or some comments that were made that were perceived as unhelpful. Some carers felt there was a “*lack of education and understanding about the medical side of an eating disorder but also the mental illness side of an eating disorder and how they interact*” (P 5). Some noted “c*omplete ignorance*” in their treating clinicians (P10) and others felt “*they were really quite clueless*” (P11). One carer summarized this difficulty as: “*I understand there’s a disconnect and lack of knowledge and misunderstanding in the community. But when you get it in the medical profession, it’s beyond frustrating* (P5)”. Some attributed this *“lack of knowledge”* (P10) to clinicians involved in the early stages of treatment seeking, which one carer termed as the “*the first point of contact*” (P12), which often included GPs, noting this was *“the main barrier”* preventing early, effective treatment for their loved one (P10).

Some carers suggested clinicians’ limited understanding of EDs was reflected in *“harmful”* (P13) or *“damaging”* (P2) comments made to their loved ones, such as telling them they *“look good”* (P13) or “*I can tell, looking at you that your BMI is fine”* (P2) when they were underweight, thereby inadvertently reinforcing ED behaviour. Another carer described an interaction where a psychologist told their loved one that it was *“perfectly okay”* (P12) to hide food instead of finishing a meal, which reportedly led to years of *“constant hiding*”, and the return of the *“eating disorder voice*”, which was a *“massive backwards step.”* This carer described how this perceived lack of insight into the way EDs present inadvertently *“undermines that sort of relationship between carer and cared person”* and parents’ authority (P12). These extracts highlight how some responses from inexperienced clinicians were perceived by carers to diminish their discernment of the ED, their relationship with their loved one, and their critical role in being there for their child in standing against the influence of the disorder.

Carers also expressed how clinicians’ limited understanding of EDs as well as the impact they have on families left them feeling unsupported. One carer stated *“we did not feel empowered or informed or hopeful”* (P7), highlighting a sense of being misunderstood or neglected in the treatment process. Other parents responded to this gap in clinician knowledge with educating themselves about ED.

“I remember thinking no one can help me” (P3)*,* noting, *“you really have to do all the work yourself. And you have to stand up and start advocating for something that you don’t know that much about”* (P3).

“an expectation that we had a baseline of knowledge that we didn’t have. We were sort of picking things up from the research that we were doing” (P12).

These extracts highlight how in the face of an absence of access to the support of experienced clinicians, these carers’ feelings of helplessness gave way to taking up the role of researching (P12) and advocacy (P3), thereby stretching their resources in their role as carer of their loved one who was living with an ED.

#### Subtheme 1.3: Co-ordination of care

Many carers also struggled with the “*clunky*” (P2) and “*disjointed*” (P4) coordination of care, with this role also falling on themselves, leading them to feel *“completely burnt out*” (P7). Indeed, one carer highlighted, *“they are meant to work together. But they don’t. They always talk about it, but it doesn’t happen”* (P11). Another suggested this disconnect was particularly pronounced *“between the medical people and the psych people*” (P5) in hospitals; this often led to disagreements over whether their loved one should be admitted or not, as they needed to be “stabilized medically” before entering the mental health unit (P5). Again, highlighting systemic challenges of siloed treatment services and un-coordinated care.

### Theme 2: Attitudes and perceptions of the ANZAED eating disorder credential

Most participants were unaware of the Credential during their loved one’s treatment journey (e.g., “I never come across that Credential before this survey”, P1), which often pre-dated the Credential (i.e., some sought treatment as early as 2013, while the Credential was first launched in 2022) [32]. These participants searched the connected website’s (https://connected.anzaed.org.au/) directory to find out if their treating clinicians were credentialed, including to check their eligibility for participation in this research. All but two of the participants had commenced treatment before the Credential was launched and as such, most carers’ reflections concerning the value of the Credential were retrospective in nature. That is, they were informed by treatment experiences with clinicians who later became credentialed after its launch.

#### Subtheme 2.1: Benefits

The carers expressed a preference for specialised ED clinicians in the care of their loved one. For some, this was positioned as “*absolutely critical*” (P8), “*definitely needed*” (P12). For example, P8 highlighted the “*cunning*” nature of EDs (i.e., “*a health disorder that [is] fighting against us all the time*”), in emphasising the need for specialised treatment. Other carers noted that if they had known their loved one was being treated by a credentialed clinician at the time, it would have instilled “*a lot more confidence*” that they were in “*the right place to go*” for effective treatment (P1). As such, they shared that, given the choice between two clinicians, “*one was credentialed, and one wasn’t, I’d definitely go for the credentialed one*” (P1). Some participants highlighted that the Credential would be particularly beneficial in training GPs, as they are often “*the first point of contact*” (P12), “*it would be so good to find GPs that had that knowledge*” (P10).

#### Subtheme 2.2: Other priorities

Although most participants viewed receiving support from a credentialed clinician as ideal, many expressed other priorities that would take precedence in their decision-making for a clinician for their loved one. For most, “*just getting the service is first priority*” (P6), noting that “*credentialing wouldn’t come up as in my top 5 sort of things*” (P6). Other important considerations included trusted referrals (P9; P6), “*working as a team*” (P3), “*the right fit*” (P6) with a clinician, with the suggestion that with a greater availability of services, “*things like the credentialing…become actually a more useful thing*” (P6). Others questioned whether “*the credentials mean much*”, highlighting that being credentialed risked being tokenistic. One parent, drawing on their experience of treatment with a credentialed clinician emphasised that effective treatment is more than being credentialed, emphasising the importance of aspects of the therapeutic relationship for both carers and their loved ones.

“[…] it does seem to be a lot of “yes, it’s on my business card”, but they don’t actually have a clue what you’re talking about […] she says she's works with eating disorders, and that but doesn't have the understanding behind it. […] This is people's feelings. This is people's mental health you're dealing with. It takes a lot more than just … I think you've got to get to an understanding of the person.” (P9).

This was echoed in the perspectives of another carer who voiced that actions speak louder than a credential: “*we just need to see more action and not just pieces of paper*” (P2), suggesting they had not yet experienced any benefits related to the Credential. Implicit in these extracts was the anxiety of being a parent living with a child with an ED in their stances for their child to be respected, heard and understood (“*understanding of the person*”; P9) and treated in ways that mobilise urgency to see tangible results of the treatment (“*more action*”; P2).

#### Subtheme 2.3: Improvements

As many carers expressed a limited understanding or awareness of the Credential when they first sought treatment, they offered some suggestions on how to extend its reach. Some carers recommended increasing the “*publicity*” (P1) around the Credential, to make it more “*visible*” to families seeking information (P4). Other carers proposed it would be “*valuable*” for the Credential to have a tiered system, whereby clinicians’ “*length of experience*” (P1) is indicated in their “*level*” of Credential or “*rating*” on the website (P3). It was noted that not only would this help carers to find clinicians with sufficient expertise in the area, but it may also “*incentivise clinicians*” to complete the necessary training (P1). Whilst the Credential consultation included with people living with an ED and carers, another carer suggested the importance of ongoing consultation—“*lived experience voices into that credentialing process*” could help clinicians to continue to develop greater understanding into the lived experience of EDs, including for family members, which may foster a greater sense of empathy (P6).

### Theme 3: Treatment experiences with credentialed clinicians

Most carers verified the credentialing status of their treating clinicians before taking part in the study, and once they had, they predominantly recalled positive experiences with these professionals. Indeed, one participant shared, “a*s it turns out, the three therapists that we’ve worked with, the clinicians that we’ve worked with, who have been good, are the three who are actually credentialed*” (P12).

#### Subtheme 3.1: Knowledge and understanding

Many participants ascribed their favorable experiences with credentialed clinicians to greater knowledge, which one participant described as a credentialed dietitian having a “*better skill set*”, “*problem solving*” ability, and *“greater insight, more understanding”* and that “*the credential did make a difference. They just seem to get it more*” (P5). P6 shared this perception, highlighting that their credentialed clinician was “*the most knowledgeable one*” their loved one had been treated by. For P5, this positive feedback was shared when comparing their experience with non-credentialed clinicians, noting there was a “*big difference*” in the quality of care they received from a credentialed vs non-credentialed dietitian. Given extensive reports (see *subtheme 2.2*) of limited clinician understanding as one of the main barriers and least helpful components of the treatment experience, this notion of detailed insight into the ED and how to approach care for a person living with an ED was emphasised as highly valued by carers.

Similarly, P12 noted that credentialed clinicians were “*able to identify and call out certain [ED] behaviours in session, attitudes or responses*”, which they feel “*kind of keeps it honest*”. Again, this sense of transparency that comes with greater experience and knowledge of EDs was greatly appreciated by carers who had previously received conflicting or unhelpful suggestions in treatment. Importantly, P12 shared that greater clinician knowledge meant that they did not have “*to educate the person that you’re speaking to and that you’re not going to be putting your loved one or your child in danger*”. Thus, it appeared that not only does greater clinician training allow for better quality care, it also has scope to reduce some of the strain and burden experienced by carers who too were greatly impacted by their loved ones’ recovery journey.

#### Subtheme 3.2: Therapeutic relationship

Many carers expressed that this greater sense of understanding in credentialed clinicians fostered stronger, more “supportive” (P5) therapeutic relationships, which helped to facilitate change. For example, P4 expressed that after completing FBT, they received parent-carer treatment from credentialed clinicians as part of a day program that they described as *“the first time that I was ever supported in the dynamic*”. With their credentialed clinicians, P4 noted, “*they absolutely made a visible display that I make the decisions*” in the family, supporting a healthier more helpful parent–child dynamic to work with the ED.

Similarly, P5 noted, “*I think the credentialed dietitian understood the impact on the rest of us in the family better*”, thus providing greater support and prioritising family involvement in treatment. Further, P5 particularly valued the credentialed clinician’s role in supervising meals as this could be inadvertently “*damaging*” to the parent–child relationship. This allowed P5 to just “*be mum*” rather than her child’s “*adversary that lingered over him 6 times a day over food*”. Finally, this carer felt that their credentialed dietitian’s “*greater understanding and insight led to her communicating better*”, allowing for effective family engagement (P5). Furthermore, P7’s current experiences of credentialed clinicians were that they “s*eem to be listening to us a bit more and respecting us a bit more*”, noting that their child “*seems to be a lot more loved. Yeah, you know what I mean, like, you know, appreciated. Yeah, rather than being told what’s wrong about him, so maybe this is more of a strength-based approach*.” These carer accounts highlight how their experiences of greater understanding came with greater support and better treatment outcomes.

## Discussion

The current study aimed to explore carers’ experiences and attitudes towards ED treatment with clinicians who hold the ANZAED Eating Disorder Credential. This included to develop an understanding into whether the predicted benefits of this new reform translated into positive treatment experiences from the perspectives of carers and to inform future improvements of the Credential. Inductive thematic analysis generated three main themes from the data, which clustered around the treatment experiences of carers prior to the launch of the Credential (Theme 1), their attitudes and perceptions of the Credential (Theme 2), and their experiences of treatment for their loved one, with credentialed clinicians (Theme 3).

Regarding attitudes and perceptions, carers viewed the Credential as vital due to the unique complexities of EDs, necessitating specialised treatment. Some carers suggested this specialised training would be particularly useful for professionals who represent the first point of contact for help seekers (e.g., General Practitioners). Importantly, in March 2024 the ANZAED Eating Disorder Credential was expanded to general practitioners [33]. Carers also shared that knowing their clinicians were credentialed would increase their confidence in treatment effectiveness. This supports qualitative predictions anticipating the Credential would facilitate early intervention and instil greater confidence [31]. This also aligns with findings in other areas of health service provision suggesting that clinician credentials enhance consumer confidence [22, 23].

Although carers viewed the Credential as important, they emphasised priorities for the clinician treating their significant other, that were not confined to the question of whether or not treating clinicians were credentialed. For example, carers identified simply accessing care as their primary focus, followed by effective teamwork and personalised treatment. This corroborates global recommendations for effective ED intervention, emphasising the need for multidisciplinary [37–40] and individualised care [11, 41].

As most carers had only discovered the Credential recent to their participation in this study, they recommended an increase in its dissemination so that families could access specialised care earlier in treatment. Carers also suggested as a future adaptation and expansion to the Credential, offering different levels of credentialing recognising different levels of training and experience. This maps onto research demonstrating how credentialling can encourage clinicians to take initiative when it comes to ongoing vocational training [21]. In line with the Credential guidelines, carers also supported the perspective that lived experience voices should be integrated into the Continuing Professional Development (CPD) training for clinicians to maintain the Credential. Carers believed this would help to ensure that clinicians possess a richer understanding of EDs from lived experience perspective as well as the basic knowledge required to treat them.

Similar to qualitative reports gathered while developing the Credential [31], carers described facing many challenges along the treatment journey, prior to securing treatment with a credentialed clinician. These included delayed diagnosis, long waitlists and unhelpful comments made by likely inexperienced clinicians. Carers also expressed that these difficulties left them, and their loved one, feeling frustrated, unheard, uninformed, and unsupported in the face of the disorder; these challenges are well documented in the literature [15, 27, 42–44]. Among these concerns, carers alluded to broader systemic issues including disjointed treatment services, insufficient funding and resources, issues that may stretch beyond the reach of the Credential.

Finally, carers recounted positive experiences with credentialed clinicians, characterised by greater perceived knowledge and insight. Many carers compared their experiences between credentialed and non-credentialed clinicians, noting that with the former, they often felt more heard, supported, and integrated in the treatment process; carers also perceived credentialed clinicians as more likely to cater to their loved one’s individual needs. It is important for credentialed clinicians to recognise that carers value treatment factors over and above whether a clinician is credentialed; this is unsurprising given the literature that supports the importance of personalised care in effective ED treatment [10, 11, 41, 45, 46].

The present study holds significant implications for the effective application of the Credential in the future. Notably, the current study illustrates how, so far, the Credential has yielded positive outcomes for individuals experiencing EDs and their families as perceived and experienced by carers. Our results suggest that, with increased publicity, families and their loved ones may acquire these benefits from the outset of treatment, allowing for much needed early intervention and co-ordinated care with effective and tailored ED treatments [47–49]. These findings also reinforce the importance of including carers and those with lived experience in every stage of treatment, from its design and planning to implementation [8]. Finally, the current research underscores the importance of training for clinicians in addressing broader systemic issues in eating disorder treatment provision including the effects of an ED on a family system, which are commonly reported by carers, including parents and partners, and other key stakeholders [31]. These also include the need for ongoing improvement, particularly in the coordination of services, and reaffirms the necessity for specialist training and supervision, which the Credential aims to provide.

Regarding its strengths, the current study is the first in depth exploration of carer experiences and perceptions of treatment with ANZAED Credentialed Eating Disorder Clinicians; as such, the results make an important contribution to bridging the research-to-practice gap when it comes to including families and carers in all facets of ED care. Another strength was the study’s inductive design that allowed carers’ experiences to guide the thematic framework, reflecting their perceptions authentically. On the other hand, the results need to be interpreted in light of this group of carers being self-selected where some of the perspectives arose from their unique eating disorder treatment experiences. In addition, marginalised groups were underrepresented and the Credential was new, suggesting that further research is needed to trace the longer term impacts of the Credential and with a more culturally and socio-economically diverse sample of carers. Furthermore, some participants retrospectively learned that their clinicians were credentialed and therefore their experiences of treatment may have been biased by this recently learned information. Future research should consider administering interviews to carers upon their loved one’s discharge from treatment to avoid any bias that may be introduced by retrospective recall. Many participants also shared their positive attitudes in comparison to their experiences with non-credentialed clinicians, suggesting that their focus on negative experiences prior to seeing a credentialed clinician may have overshadowed any concerns with their treatment experiences with a credentialed clinician. Due to the complex nature of eating disorders and the time constraints of the interview, carers at times also reflected on some of their loved one’s treatment experiences more generally (e.g. an inpatient admission) rather than focusing on individual treatment providers and therefore it was difficult to ascertain the credentialing status of all clinicians involved in their loved one’s treatment. However, all carers needed to have had experience with their loved one receiving treatment from at least one credentialed clinician whom they were specifically asked to reflect upon during the interview. We also didn’t explicitly ask all participants who were interviewed about the training and/or experience of the non-credentialed clinician they had treatment experiences with and so, are unable to determine if they had specific expertise in eating disorders. Therefore, further research is needed to explore carer experiences more comprehensively, including in exploring the longitudinal impacts of the Credential treatment experiences.

In conclusion, this study found that the carers perceived treatment from Credentialed Eating Disorder Clinicians as substantially helpful in facilitating supportive and effective intervention for their significant other. However, many expressed that several barriers to effective treatment persisted in the broader ED treatment landscape that need to be further addressed. There is also a need to further understand the impacts of the rollout of the Credential over time before the full potential benefit of the Credential may be realised. Carers also suggested that increased publicity could help to broaden the Credential’s scope and capacity to overcome some of the barriers they faced in their treatment experiences. Therefore, continued consideration is needed to ensure the Credential can help to improve intended outcomes for individuals living with EDs and their carers.

## Supplementary Information

Additional Files

## Data Availability

The datasets used and/or analysed during the current study are not publicly available. They may be available from the corresponding author upon reasonable request and in accordance with Human Research Ethics permissions. Permission for the data to be made publicly available is not sought as it would identify participants.

## Abbreviations

ANZAED: Australian & New Zealand Academy for Eating Disorders
ARFID: Avoidant/restrictive food intake disorder
CBT: Cognitive behavioural therapy
CPD: Continuing professional development
ED: Eating disorder
EDFA: Eating disorders families Australia
EDQ: Eating disorders Queensland
EDV: Eating disorders Victoria
FBT: Family based therapy
NEDC: National eating disorders collaboration
OSFED: Other specified feeding or eating disorder
