# Supplementary material for: “The credential did make a difference”: eating disorder treatment with ANZAED credentialed clinicians: carer perspectives
Source: J Eat Disord. 2025 Aug 28;13(Suppl 1):193. doi: 10.1186/s40337-025-01383-y (PMC12395635; doi:10.1186/s40337-025-01383-y)
Supplement: Supplementary file 1 — Additional Files [file 40337_2025_1383_MOESM1_ESM.pdf]

Additional File 1. Carer Survey Questions

Thank you for agreeing to participate in this study!

To begin with, please create your **unique participant ID** below.

Your ID is: first two letters of your mother's/caregiver's name written in uppercase, followed by last four digits of your phone number (e.g., if your mother's name is Sarah, and your phone number is +61 1 2345 6789, your ID would be SA6789).
(If you do not want to participate in the interview, this ID will be removed from your survey)

ID: _______________

1. **Demographics**

   Please answer the following general questions about you.

1.1. Which option best describes your gender?

- Man
- Woman
- Other (Please specify)
  - Free text response
- Prefer not to say

1.2. What is your age in years?

- Free text response

1.3. What is your cultural and ethnic background?

- Oceanian (e.g., Australian, New Zealand, Polynesian)
- European (e.g., British, Irish, Western European, Northern European)
- Asian (e.g., Chinese, Japanese)
- Middle Eastern (e.g., Arab, Egyptian)
- North American (e.g., United States of America, Canadian)
- Central and South American (e.g., Hispanic)
- Sub-Saharan (e.g., Central and West African, Southern and East African)
- Prefer not to say

1.4. In which Australian state or territory do you currently reside?

- Australian Capital Territory
- New South Wales
- Northern Territory
- Queensland

- South Australia
- Tasmania
- Victoria
- Western Australia
- None

1.5. Which geographical setting best describes your primary residential location?

- Metropolitan
- Regional
- Rural
- Remote

1.6. What is your marital status?

- Single
- Married/living as married
- Separated, divorced or widowed

1.7. What is your current employment status? Please select all that apply.

- Currently studying
- Employed full-time
- Employed part-time/casually
- Full time home duties/caring for children
- Presently not employed, seeking employment
- Presently not employed, NOT seeking employment
- Recovering from illness or injury
- Other (Please specify)
    - Free text response

1.8. What is the highest level of education you have completed to date?

- Prior to Year 10
- Year 10 high school
- Year 12 high school
- Trade certificate apprenticeship
- University or Tertiary degree or College diploma

2. Eating Disorder History

2.1. Has your loved one ever experienced symptoms of an eating disorder? (For example, purging, restricting food intake)

- They are currently experiencing eating disorder symptoms
- They have previously experienced eating disorder symptoms
- They have never experienced eating disorder symptoms

2.2. In what year (approximately) did your loved one first start experiencing symptoms of an eating disorder? Please specify:

- Free text response

2.3. Has your loved one ever been diagnosed with an eating disorder? If yes, please select all that apply.

- No, they have not
- Anorexia Nervosa
- Bulimia Nervosa
- Binge Eating Disorder
- Other Specified Feeding or Eating Disorder (OSFED)
- Pica
- Rumination Disorder
- Avoidant/Restrictive Food Intake Disorder (ARFID)
- Unspecified Feeding or Eating Disorder
- Other (Please specify)
  - Free text response

Question only shown to participants who did not select "No, they have not":

2.4. In what year (approximately) was your loved one first diagnosed with an eating disorder? Please specify.

- Free text response

3. Treatment History

3.1. Has your loved one ever received treatment from a professional or specialist for an eating disorder?

- Yes, they are currently receiving treatment for an eating disorder
- Yes, they received treatment in the past but not currently

- No, they have never undergone treatment for an eating disorder

Questions 3.2-3.13. only shown to participants who answered “Yes”:

3.2. Please indicate which professionals or specialists your loved one received treatment from. Please select all that apply.
   - Dietitian
   - General Practitioner
   - Occupational Therapist
   - Psychiatrist
   - Psychologist
   - Psychotherapist
   - Social Worker
   - Other (Please specify)
     - Free text response

3.3. Which geographical setting best describes the location your loved one received most of their eating disorder care?
   - Metropolitan
   - Regional
   - Rural
   - Remote

3.4. From the time your loved one began to seek treatment, how long did they have to wait to access it? (To the nearest month).
   - Free text/months

3.5. Please select the type of treatment(s) your loved one has undertaken for the eating disorder. You can select more than one option.

   Please RANK the treatments in order of DURATION with 1 indicating the longest treatment then 2, 3, 4 etc. If your loved one has only experienced one treatment for an eating disorder, just mark 1 in the box.
   - Individual Therapy
   - Family Based Therapy
   - Inpatient Therapy
   - Group Therapy

- Nutritional Counselling/Physical Health CounsellingMedical/Psychiatric Treatment
- Other (Please specify)
    - Free text response

3.6. Please select which of the following "Individual Psychological Therapies" your loved one has experienced (if known). Please select all that apply.

- Cognitive Behavioural Therapy (CBT)
- Motivational Interviewing (MI)\
- Behaviour Therapy
- Dialectical Behaviour Therapy (DBT)
- Narrative Therapy
- Mindfulness Based Therapy
- Acceptance and Commitment Therapy (ACT)
- Psychotherapy
- Psychodynamic Therapy
- Other (Please specify)
    - Free text response
- I do not know what type of therapy my loved one received

3.7. Please select which of the following "Family Therapies" your loved one has experienced. Please select all that apply.

- Maudsley Family Based Treatment (FBT)
- Other Family Therapy (Please specify)
    - Free text response

3.8. Please select which of the following "Inpatient Therapies" your loved one has experienced. Please select all that apply.

- Eating Disorders Inpatient Treatment
- General medical ward
- Other (Please specify)
    - Free text response

3.9. Please select which of the following "Group Therapies" your loved one has experienced. Please select all that apply to you.

- Dialectical Behaviour Therapy
- Skills Focused Groups
- Education Focused Group

- Eating Disorders Group

3.10. Please select which of the following “Nutritional Counselling Services” your loved one has experienced. Please select all that apply.

- By an accredited Practising Dietitian
- Nutritionist
- Naturopath
- Other counselling

3.11. Please select which of the following “Physical Health Counselling Services” your loved one has experienced. Please select all that apply.

- Personal fitness trainer
- Other physical health counselling (Please specify):
    - Free text response

3.12. Please select which of the following “Medical/Psychiatric treatments” your loved one has experienced. Please select all that apply.

- Medications (please specify type of medications)
    - Free text response
- Other (please specify)
    - Free text response

3.13 Please share with us any other treatment interventions for eating disorders that your loved one has tried?

- Free text response

4. Experience using *Find a Treatment Provider Search* Directory

4.1. Have you ever accessed the *Find a Treatment Provider Search* Directory which contains a registry of all Credentialed Eating Disorder Clinicians: https://connected.anzaed.org.au/treatmentproviders/?

- Yes
- No
- I’m not sure

Questions 4.3-4.8 only shown to participants who answered “Yes”:

4.2. Do you have a strong preference for seeing a particular type of clinician?

- Yes

- No

Question 4.2.1 only shown to participants who answered "No":

4.2.1 Can you please elaborate on the reasons why you don't have a strong preference?

4.2.2 Is it important for you to find a clinician based on any of the following? Please select all that apply.

- Demographic characteristics (e.g. age, sex, ethnicity)
- Psychological characteristics (e.g. respect for your beliefs and cultural values)
- Physical characteristics (clinician weight and body shape)

4.3. Did the search filters assist in this process? Specifically in relation to the following aspects of the treatment provider:

4.3.1. Location

- Yes
- No

4.3.2. Gender

- Yes
- No

4.3.3. Areas of interest (e.g., child and adolescence health, LGBTIQ health, Aboriginal and Torres Strait Islander health)

- Yes
- No

4.3.4. Language spoken

- Yes
- No

4.4. Are there any other filters you would like us to consider developing to make your search more customisable and tailored to your needs?

- Free text response

4.5. Did the search directory always display potential service providers in response to your search criteria?

- Yes
- No
- I'm not sure

4.6. Did you use the clinician profile function?

- Yes
- No
- I'm not sure

*Question 4.6.1 only shown to participants who answered "Yes":*4.6.1 Was the clinician profile function helpful?

  - Free text response

4.7. Did the Find a Treatment Provider Directory reduce the amount of time it took you to find a clinician?

- Yes
- No
- I'm not sure

4.8. Do you feel that the Find a Treatment Provider Directory reduced the waiting time to access treatment?

- Yes
- No
- I'm not sure

5. ANZAED Credentialed Eating Disorder Clinician treatment experience

5.1. To your knowledge has your loved one ever received eating disorder treatment from a Credentialed Eating Disorder Clinician? (If unsure, please check by looking them up on the Find a Treatment Provider Directory which contains a registry of all Credentialed practitioners: https://connected.anzaed.org.au/treatmentproviders/)

- Yes, they are currently receiving treatment from a Credentialed clinician
- Yes, they received treatment in the past from a Credentialed Eating Disorder Clinician, but I am no longer seeing them
- No
- I'm not sure

Questions 5.2-5.8 only shown to participants who answered "Yes":

5.2. Please indicate which Credentialed Eating Disorder Clinician/s your loved one received treatment from. Please select all that apply.

- Counsellor
- Dietitian
- General Practitioner
- Mental Health Nurse
- Nurse Practitioner
- Occupational Therapist
- Psychiatrist
- Psychologist
- Psychotherapist
- Social Worker

Please answer the following questions with reference to the Credentialed Eating Disorder Clinician your loved one saw for the longest period of time.

5.3. From the time your loved one began to *seek* treatment from a Credentialed Eating Disorder Clinician, how long (approximately) did they have to wait to see them? (to the nearest month)

- Free text response/months

5.4. How long (approximately) have they received treatment from a Credentialed Eating Disorder Clinician? (to the nearest month)

- Free text response/months

5.5. Are they still receiving this treatment?

- Yes
- No

5.6. How did you find a Credentialed Eating Disorder Clinician?

- connected website
- Referral by GP or other health professional
- Referral by family, friends, carers or other people who are currently or have previously been diagnosed with an eating disorder
- Independent advertising (websites, clinic brochures/posters, etc.)
- Social Media (Facebook, Instagram, Twitter)
- Webinar/Seminar
- Podcast
- Other (Please specify)
    - Free text response

5.7. Which geographical setting(s) best describes the location your loved one received care from a Credentialed Eating Disorder Clinician?

- Metropolitan
- Regional
- Rural
- Remote

5.8. Which of the following treatment format(s) did your loved one use to receive care from a Credentialed Eating Disorder Clinician? Please select all that apply.

- In-person
- Telehealth (online)
- Telephone
- Outpatient facility
- Inpatient facility
- Other (Please specify)
    - Free text response

10. Attitudes Toward the ANZAED Eating Disorder Credential

Whether or not you have your loved one received eating disorder treatment from a Credentialed Eating Disorder Clinician, please take the time to answer the following questions.

10.1. After you have read each question, please drag the slider to the point that most accurately reflects the extent to which you agree with the following statements:

10.1.1. In terms of the success of therapy, it makes no difference whether they receive treatment from a Credentialed or non-credentialed clinician.

Strongly Disagree Strongly Agree

10.1.2. I would prefer for them receiving treatment from a Credentialed Clinician over a non-credentialed clinician.

Strongly Disagree Strongly Agree

10.1.3. I place greater trust in the advice of a Credentialed Eating Disorder Clinician than a non-credentialed clinician.

Strongly Disagree Strongly Agree

10.1.4. I value the existence of a credential that recognises the expertise and training of clinicians and requires a commitment to engagement in continual professional development and supervision specific to the treatment of eating disorders.

Strongly Disagree Strongly Agree

10.1.5. The Credential will make it easier for people with an eating disorder to readily access specialised care.

Strongly Disagree Strongly Agree

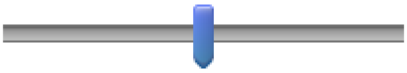

10.1.6. The Credential will improve the health outcomes of eating disorder patients.

Strongly Disagree Strongly Agree

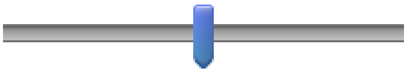

10.2. The following questions ask about your experiences of the connected website (https://connected.anzaed.org.au). This website is designed to assist with access to Credentialed Eating Disorder Clinicians.

10.2.1. Have you used this website before?

- Yes
- No

Questions 10.2.2.-11. shown only to participants who answered "Yes":

10.2.2. How did you first hear about the connected website?

- From a health care professional
- From a friend/peer
- On the internet
- Other (please specify):

After you have read each question, please drag the slider to the point that most accurately reflects the extent to which you agree with the following statements:

The connected website is easy to navigate and useful for locating eating disorder treatment service providers.

Strongly Disagree Strongly Agree

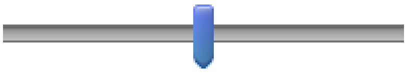

The information in clinician profiles was detailed and clear such that I felt confident to select a clinician who was the 'right fit' for my loved one.

Strongly Disagree Strongly Agree

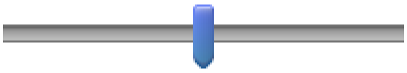

The search filters helped me to easily identify a clinician who was the 'right fit' for my loved one.

Strongly Disagree Strongly Agree

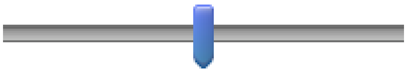

I felt that I could trust the information presented on the connected·ed website.

Strongly Disagree Strongly Agree

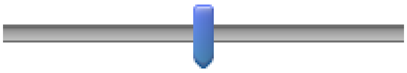

The connect·ed website used appropriate, respectful and inclusive language.

Strongly Disagree Strongly Agree

	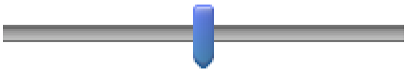

I would recommend the connect·ed website to other patients, carers and families who are seeking specialised treatment for an eating disorder.

Strongly Disagree Strongly Agree

	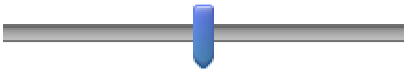

11. Perceived barriers to seeking and accessing treatment questionnaire (Hamilton et al., 2022).

Please respond on the following questions using your own experience and perspective on barriers to seeking and accessing treatment.

Please rate how each of the following barriers affected your loved one's access or use of outpatient or inpatient eating disorder treatment:

11.1. Cost (e.g. cost of service, travel expenses, time off work)

No impact at all		*Moderately impactful*		*Extremely impactful*
1	2	3	4	5

11.2. Stigma associated with eating disorder (e.g., did e.g., did not feel comfortable disclosing my condition to my GP, guilt or shame associated with my symptoms, fear of judgement from family/social group)

No impact at all *Moderately impactful* *Extremely impactful*

1 2 3 4 5

11.3. Inaccessible treatment (e.g., distance to treatment facility, long waitlist to get into treatment facility)

No impact at all *Moderately impactful* *Extremely impactful*

1 2 3 4 5

11.4. Social/work barriers (e.g., family obligations, work commitments)

No impact at all *Moderately impactful* *Extremely impactful*

1 2 3 4 5

11.5. GP/health eating disorder knowledge (e.g., GP/physician did not recognise the eating disorder)

No impact at all *Moderately impactful* *Extremely impactful*

1 2 3 4 5

11.6. Personal eating disorder knowledge (e.g., not knowing about treatments for eating disorders or where to find them)

No impact at all *Moderately impactful* *Extremely impactful*

1 2 3 4 5

11.7. Other, please specify: _________

No impact at all *Moderately impactful* *Extremely impactful*

1 2 3 4 5

© **This survey is copyrighted to Janet Conti, Western Sydney University, and is not to be used or distributed without permission.**

FINISH

Thank you very much for your interest and participation in our research.

If you wish to be contacted for an **interview**, please provide your contact details below (these will be stored separately from your survey responses).

Email address: _________________

Alternative email address (if applicable): _________________

Phone number: _________________

Also, if you wish to receive **a summary of study findings**, please provide your contact details below (these will be stored separately from your survey responses).

Email address: _________________

Alternative email address (if applicable): _________________

If you have any questions, concerns, comments or would like to participate in any further studies into eating disorders and their treatment, please contact Dr. Janet Conti (email: j.conti@westernsydney.edu.au) or Professor Phillipa Hay (email: p.hay@westernsydney.edu.au)

If you find yourself experiencing any distress please contact your local GP, therapist or eating disorder services.

The following services also offer counselling support in Australia:

The Butterfly Foundation - 1800 334 673 or online (www.thebutterflyfoundation.org.au)

Beyond Blue: 1300 22 4636

Lifeline: 13 11 14

APS Find a Psychologist Webpage: http://www.psychology.org.au/FindaPsychologist

ANZAED Find a Treatment Provider Directory:
https://connected.anzaed.org.au/treatmentproviders/

The ethical aspects of this study have been approved by the Western Sydney University Human Research Ethics Committee. If you have any complaints or reservations about any ethical aspect of your participation in this research, you may contact the Human Ethics Officer (telephone (02) 4736 0493; email humanethics@westernsydney.edu.au). Any complaint you make will be treated in confidence and investigated, and you will be informed of the outcome.

Additional File 2. Comparing the Carer Characteristics of Participants who Completed the Survey and Interview vs. Participants who Completed the Survey Only

Carer characteristics	Survey & Interview (n = 13)	Survey only (n = 55)	Statistical testing (n =68)	
	Median (IQR)		Z^1	P
Age (years)	52 (47- 53.5)	50 (46-53)	-1.173	.241
Length of treatment (months)	24 (11-25.5)[2]	18 (12-36)[3]	-.213	.863
	n (%)		χ^2, df	P
Identified gender				
Female	12 (92.3%)	53 (96.4%)	.410, 1	.477[4]
Location of primary residence				
Metropolitan	10 (76.9%)	41 (74.5%)	.032, 1	.585[4]
Marital Status				
Married/Living as married	10 (76.9%)	43 (78.2%)	.010, 1	.590[4]
Employment status				
Employed part-time, full-time, or casually	11 (84.6%)	41 (74.6%)	.593, 1	.357[4]
Level of education				
Tertiary	12 (92.3%)	39 (70.9%)	2.568, 1	.101[4]
Currently experiencing symptoms	10 (76.9%)	42 (76.4%)	.002, 1	.639[4]
ED Diagnosis				
Anorexia Nervosa	11 (84.6%)	49 (89.1%)	.203, 1	.478[4]

Note: Cell counts for some responses have been suppressed to protect confidentiality of sensitive/identifying information. [1]Mann-Whitney U test [2]This analysis was missing data, n = 10 [3]This analysis was missing data, n = 11 [4]Fisher exact test used as some cell counts were <5

Additional File 3. Carer Interview Questions

A <u>selection of questions</u> will be used with each participant and will scaffold between:

- **Experience (e.g. Can you tell me about …?)**
- **Meaning (What does … mean to you?)**

1. What has made you interested in wanting to participate in this research today?

2. Can you tell me which family member has been affected by an eating disorder?
   Prompts:
   i. What diagnosis did your loved one receive?

3. Aside from yourself, was there anyone else who was looking after your loved one / [insert name]?

4. Can you tell me about the development of your loved one’s eating disorder?
   Prompts:
   i. Can you tell me about when you first noticed something was wrong? How old were they?
   ii. How do you feel the eating disorder affected your family? OR in what way did the eating disorder affect your family?
   iii. How old was your loved one when they first sought help/diagnosis/treatment for an eating disorder?

5. Can you please tell us about your loved one’s experiences in accessing and receiving care for their eating disorder?

   Prompts:
   i. When your loved one first sought help, did they make the decision on their own or did someone else?
   ii. What contributed to the decision to seek care?
   iii. Did you or your loved one experience any barriers to accessing care? If so, can you please tell me about them.
   iv. Were there any resources, people, or organisations that assisted you in accessing care for your loved one? If so, can you please tell me about them.

6. What treatment(s) did they receive?

   Prompts:
   i. How long have they been in treatment? Different therapists? Inpatient?

7. Has your loved one accessed care from clinicians who are credentialed?
   Prompts:
   i. When did they first start seeing [name of clinician(s)]?
8. When reflecting on their time receiving care, what did you find most helpful in your interactions with clinicians, treatments approaches, etc.
   Prompts:
   i. What worked well? Why was that?

9. When reflecting on their time receiving care, what did you find the least helpful in your interactions with clinicians, treatment approaches etc.
   Prompts:
   i. What didn't work well? Why was that?

10. If your loved one accessed treatment from a clinician who is credentialled, can you please tell us about that experience? [don't need to ask if covered above]

    Prompts:

    i. Was it different from other treatment experiences and in what way?

11. Were you and your family ever included in therapy?
    Prompts:
    i. Was this helpful or not? Why and why not?
    ii. What was the communication like between you and the clinician?

12. At any time in the treatment journey, were you referred to other professionals e.g. dietitians, psychiatrists etc?
    Prompts:
    i. Did you have to find these professionals on your own? If so, how did you go about doing this? (Did you experience any barriers along the way?)
    ii. Did your credentialed clinician involve other professionals in the treatment of your loved one (e.g., dietitian, psychiatrist, paediatrician, social worker)?
    iii. Were other professionals credentialed? Was this discussed or not?

13. If your loved one left (discontinued) treatment at any time, can you tell us about that experience/s?

    Prompts:

    i. Was it from a credentialled or non-credentialled clinician, or both?
    ii. Was it an in-patient, out-patient, day-patient, or other?
    iii. Did they leave on their own accord or was their treatment ended for another reasons (e.g. their progress/reduction in eating disorder symptoms?)

14. What did you value most about treatment?

15. What do you think your loved one valued the most about their experiences receiving treatment?

16. In what way did treatment impact them the most? What helped? What was least helpful?

17. Do you know much about the credentialing of eating disorder treatment providers? If so, how did you first hear about it?

18. What are your views on the credentialing of clinicians? Do you think it is important?

Prompts:

i. Would you prefer to receive care from a credentialed clinician rather than a non-Credentialed clinican? If so, why?

19. If you used the ConnectED website, can you tell us about that experience?

Prompts:

i. Is your experience what you expected? If not, how did the experience differ from your expectations?
ii. In particular, can you tell me about your experience of using the Find a Treatment Provider search directory?
iii. If you could change anything about the Find a Treatment Provider Directory what would it be?

20. Based on what you know now, what would you tell a person who is wanting to seek specialised treatment for eating disorders? What might be important for them to know?

21. Based on your experiences, what advice would you offer a clinician so they can improve their service for carers and people with eating disorders?

22. Is there anything ANZAED could do to improve things?

23. Is there anything else that we have not covered today that you think might be important to mention?

24. What is the take home message you want us to have?

Additional File 4. Researcher Positioning Statements

Each author is a clinician who has worked with individuals experiencing EDs, and Janet Conti and Phillipa Hay are also experienced researchers in the field. It is also important to note that Honor Sinclair was not involved in the interview process, limiting her interpretations to the collected responses alone. Honor Sinclair is a female, Anglo-Australian provisional psychologist and has completed a Master of Clinical Psychology. She has some limited experience providing therapy for people living with an ED as secondary presenting concerns. Janet Conti is a female, Anglo- Australian clinician and researcher who has over 30 years of experience providing therapy for people living with an ED. She started working in the field as a dietitian then as clinical psychologist and researcher. Philippa Hay is a psychiatrist and researcher. Madalyn is a psychologist, a Credentialed Eating Disorder Clinician with clinical experience working with adults with EDs, and a research officer.

Additional File 5. Exemplar Data Extracts for Themes Identified from Carers Semi-Structured Interviews

Themes	Sub-Themes	Exemplar Extracts
Theme 1: Treatment Experiences Prior to the Launch of the Credential	Subtheme 1.1 Treatment Access	*P5: " the lack of resourcing in the system, you know, like to be in emergency because my kids got these heart issues and literally having someone stand there and say, "we can't admit him". they checked five hospitals. No beds in five hospitals. And that was just gut wrenching. it felt incredibly hopeless. And it was frightening. Because you know, what happened if he had a cardiac episode while I was at the shops or something? that was the worst bit, just being told that the system couldn't help us. That was just horrendous, horrendous. he couldn't get medical clearance to go to school, and he couldn't get well till he got a bed to get refed to be well enough to go back to school. And so, you end up in this holding pattern where life is just not progressing."*<br><br>P13*: "the waiting list is really, really, really long and the roots of the eating disorder grow deeper and deeper and deeper in the meantime"*<br><br>P7: *"We did like 13 referrals before we got on to a psychologist and dietician who were able to take us."*<br><br>P8: *"I'm angry, I'm angry that I was seeing something that nobody else could see and like, why didn't we get on to it quicker?"*<br><br>P3: "*They would say her BMI is too low there's no point in engaging in conversation with her. So you really don't know what to do. You can't get into FBT. You can't get a psychologist."*
Theme 1: Treatment Experiences Prior to the Launch of the Credential	Subtheme 1.2 Knowledge and Understanding Prior to the Launch of the Credential	P2*: "I can tell, looking at you that your BMI is fine". And I was like, "ohhhhhhh", it was so damaging. And then he said, I don't think he really understood why we were there, and he said, "keep going to your psychologist", and I was like, "no, I just want you to tell me, does she need to go to hospital?" It was just terrible. So yeah, I think he had no idea."*<br><br>*P5: "lack of education and understanding about the medical side of an eating disorder but also the mental illness side of an eating disorder and how they interact."*<br><br>*P10: "complete ignorance"*<br><br>*P11: "they were really quite clueless"*<br><br>P5: *"I understand there's a disconnect and lack of knowledge and misunderstanding in the community. But when you get it in the medical profession, it's beyond frustrating"*

		P12: "*My daughter was telling her how she sometimes hid food and sometimes didn't want to finish her food. And the psychologist said, "that's perfectly okay, because it probably means that you just don't want to". And so we had a we had a couple of years of like just constant food hiding and not being able to finish because of that, and I knew I knew as soon as she, I didn't take it to that session, but I knew as soon as she come home because the the eating disorder voice was back, and she walked in the door, and I was talking to the eating disorder. And And that that like that hadn't been there for a long time. And so that was a massive backwards step."*<br><br>P13: *"They really say triggering things as well like, for example, that she came in for a third time and then "oh you look good today."*
Theme 1: Treatment Experiences Prior to the Launch of the Credential	Subtheme 1.3 Co-ordination of Care	P5: *"the other difficulty I found with eating disorders, was the disconnect often between the medical people and the psych people. So, we had one admission where psych was saying, "he has to be admitted. He's really bad." And he needed to be stabilised medically before he could go into the psych unit and medical were saying "oh, we don't think he's that bad. He's fine. He can go home". And psych really had to push to get him admitted."*<br><br>P2: "*I just felt like she was here really messed around. And her treatment was so clunky like saying private and then going to CYMS."*<br><br>P11: "*they are meant to work together. But they don't. They always talk about it, but it doesn't happen. I don't know why."*<br><br>P7: *"So I'm still responsible for all of this administration, which is stuff that people used to get paid to do...". "I'm burnt out, I'm completely burnt out. I'm completely burnt out and I know that this is, oh now I am crying.."*
Theme 2: Attitudes and Perceptions of the ANZAED Credential	Subtheme 2.1: Benefits	P8: *"I think it's critical. Yeah. absolutely critical. It is such a unusual area of health, and it's so complicated...it's so cunning." "we were dealing with the health disorder that was fighting against us all the time".*<br><br>P1: "*if I was in the situation where I was selecting a psychologist. There were 2 available. One was credentialed, and one Wasn't in eating disorders. I'd definitely go for the credentialed one."*<br><br>*P10: "it would be so good to find GPs that had that knowledge".*<br><br>*P12: "I think everyone, but probably especially GPs as perhaps the first, possibly the first point of contact, and the first kind of opportunity to maybe have an uncomfortable conversation with somebody."*

Theme 2: Attitudes and Perceptions of the ANZAED Credential	Subtheme 2.2 Other Priorities	*P6: "credentialing wouldn't come up as in my top 5 sort of things, I mean finding a good, a good therapist definitely. You know someone who's going to be there consistently, and you know who can establish a good rapport and who has the appropriate training, is definitely, you know, like been a key for us... it wouldn't be top of the list, you know. It's finding someone who's who's the right fit and who's available is [important]."*<br><br>*P9: "it does seem to be a lot of "yes, it's on my business card". But they don't actually have a clue what you're talking about.".. "I'm not sure the credentials mean much."*<br><br>*P2: "I just think we just need to see more action and not just pieces of paper."*<br><br>*P6: "it was just about just getting the service is first priority, so I was glad that she [professional] was credentialed, but it was probably the recommendation from PEDS was more important, rather than knowing she was credentialed externally".*<br><br>*P3: it was important that they had the credentials, but it was also important that I knew that they sort of worked as a team."*
Theme 2: Attitudes and Perceptions of the ANZAED Credential	Subtheme 2.3 Improvements	P4: *"the more visible it is, is really important for families when they're seeking information"*<br><br>P1*: "publicity side and getting their name out there... I sit in the waiting room and read all the notice boards "(P1)*<br><br>P3: *"having sort of a number or a rating will be valuable, because it might also incentivize clinicians to do that work. I think that's really important, and that the levels are very high for the credentialing and the expertise that they have to have."*<br><br>P6: *"Well, I mean I don't know what what's involved in the credentialing, in terms of getting any sort of lived experience voices into that credentialing process".*

Theme 3: Treatment Experiences with Credentialed Clinicians	Subtheme 3.1: Knowledge and Understanding	P5: "*so we had an earlier dietitian who wasn't credentialed, and then subsequent to that, big difference. Insight. She had greater insight, more understanding. I think her communication. I don't know if that was temperament, though, or whether just her understanding and insight led to her communicating better...*" *"the credential did make a difference. They just seem to get it more. And I think the other thing they get is, the other difference that's just occurred to me, I think the credentialed dietitian understood the impact on the rest of us in the family better...And I think she got how I didn't want to be the bad guy anymore with the food. She was very supportive of us, you know, having support workers do meal supervision, because I wanted to be mum. I didn't want to be this adversary that lingered over him 6 times a day over food, and then, 3 or 4 times a day on top of that with supplement drinks. Ten battles a day was just exhausting, and it damaged our relationship."*<br><br>P12: *"just being able to identify and call out certain behaviors in session, attitudes or responses, kind of keeps it honest."*<br><br>P12*: "So yeah, the I think the credentialing side of things is really important... Because you've got a, it gives you a level of confidence that that that sort of baseline of knowledge is there, and that you're not going to be having to educate the person that you're speaking to and that you're not going to be putting your loved one or your child in danger."*
Theme 3: Treatment Experiences with Credentialed Clinicians	Subtheme 3.2: Therapeutic Relationship	P5: "*And I think she got how I didn't want to be the bad guy anymore with the food. She was very supportive of us, you know, having support workers do meal supervision, because I wanted to be mum. I didn't want to be this adversary that lingered over him 6 times a day over food, and then, 3 or 4 times a day on top of that with supplement drinks. Ten battles a day was just exhausting, and it damaged our relationship*."<br><br>P4: *"I did parent-carer treatment with [credentialed clinician]. She's also a social worker. So she put together the parent-carer sessions, which were a part of the treatment. We did FBT over a 6 month period. That was the first time that I was ever supported in the dynamic."*<br><br>P7: "I suppose the thing is now, people just seem to be listening to us a bit more and respecting us a bit more...." "probably feel more supported..." "yeah, and he seems to be a lot more loved. Yeah, you know what I mean, like, you know, appreciated. Yeah, rather than being told what's wrong about him, so maybe this is more of a strength based approach."
